# Supplementary material for: Primate anterior insular cortex represents economic decision variables proposed by prospect theory
Source: Nat Commun. 2022 Feb 7;13:717. doi: 10.1038/s41467-022-28278-9 (PMC8821715; doi:10.1038/s41467-022-28278-9)
Supplement: Supplementary file 1 — Supplementary Information [file 41467_2022_28278_MOESM1_ESM.pdf]

Primate anterior insular cortex represents economic decision variables proposed by prospect theory

You-Ping Yang[1,2,†], Xinjian Li[2,3,†], Veit Stuphorn[1,2,3]*

[1]Department of Psychological and Brain Sciences, Johns Hopkins University, 3400 N. Charles St., Baltimore, MD 21218-2685, USA

[2]Zanvyl Krieger Mind/Brain Institute, 3400 N. Charles St., Baltimore, MD 21218-2685, USA

[3]Department of Neuroscience, Johns Hopkins University School of Medicine, 3400 N. Charles St., Baltimore, MD 21218-2685, USA

***Corresponding author:**

Veit Stuphorn
Johns Hopkins University
338 Krieger Hall
3400 N. Charles St.
Baltimore, MD 21218
USA
Tel: (410) 516-7964
Fax: (410) 516-8648
Email: veit@jhu.edu

Supplementary Information

Supplementary Figures 1-8

Supplementary Tables 1-2

Supplementary Fig. 1

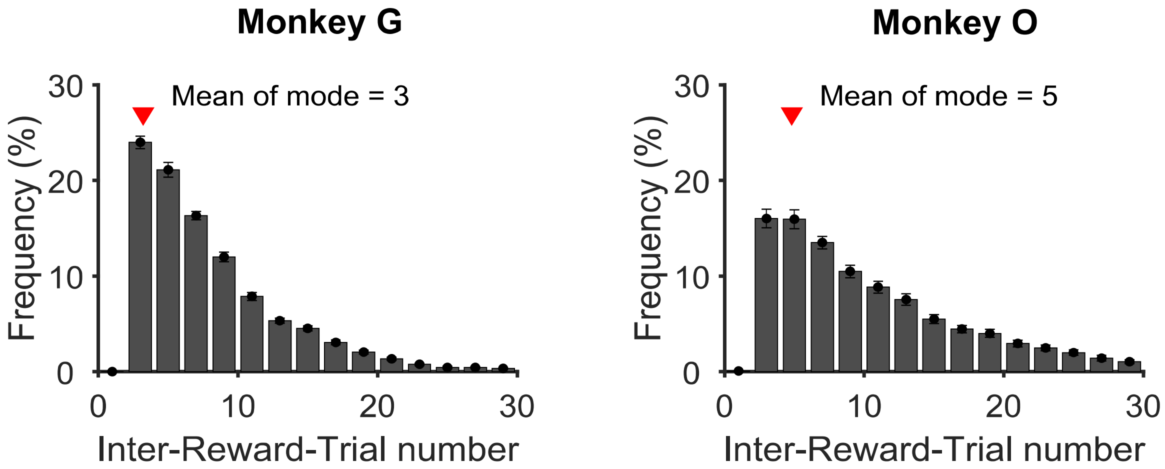

Supplementary Fig. 1. Inter-Reward-Trial number for each monkey.

Distribution of trial numbers that monkeys needed to accumulate the necessary 6 tokens for a standard fluid reward. Red triangle indicates the average mode of the number of trials to get the reward. n = 37 sessions for both monkey G and monkey O. Data are presented as mean values ± SEM

Supplementary Fig. 2

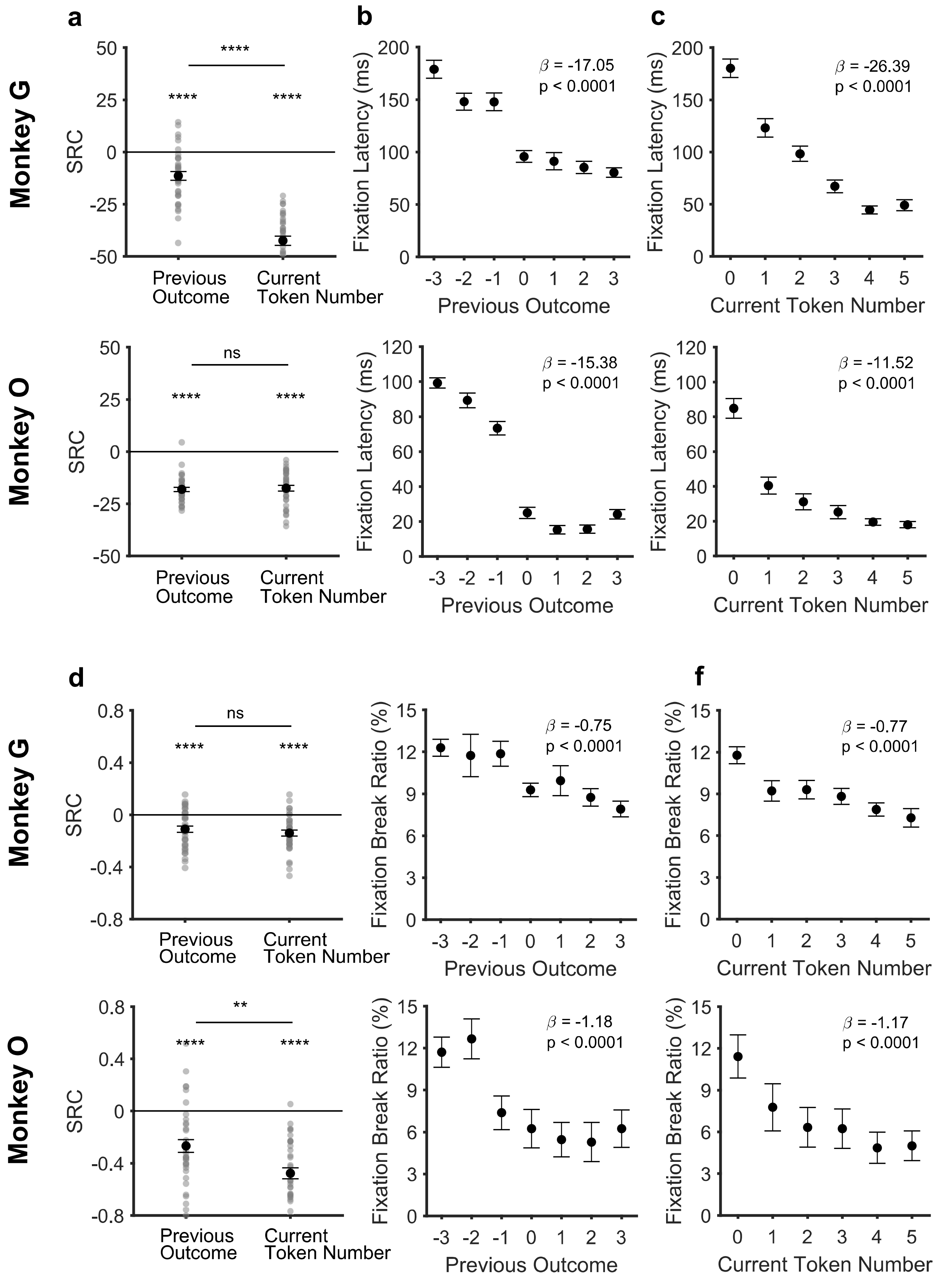

Supplementary Fig. 2. Effect of current token asset and token outcome history on fixation latency and fixation break ratio.

a. Standardized regression coefficients (SRCs) for fixation latency (latency to fixate on the center point at the beginning of the trial before the token cue appears) as a function of previous outcome (token change in the last trial) and current token number.

b. Fixation latency as a function of previous outcome. Regression coefficient (β) between fixation latency and the token number won or losses of the previous trial.

c. Fixation latency as a function of current token asset. Regression coefficient (β) between fixation latency and the start token number of the current trial.

d. Standardized regression coefficients (SRCs) for fixation break ratio (failure to hold fixation on the center point long enough for token cues to appear) as a function of previous outcome (token change in the last trial) and current token number.

e. Fixation break ratio as a function of previous outcome. Regression coefficient (β) between percentage of trials with fixation breaks and the token number won or losses of the previous trial.

f. Fixation break ratio as a function of current token asset. Regression coefficient (β) between percentage of trials with fixation breaks and the start token number of the current trial.

n = 37 sessions for both monkey G and monkey O. Data are presented as mean values ± SEM. ns = not statistically significant (i.e. $p > 0.05$), ** = $p < 10^{-2}$, *** = $p < 10^{-3}$, **** = $p < 10^{-4}$ for 1-sided t-test or paired t-test.

Supplementary Fig. 3

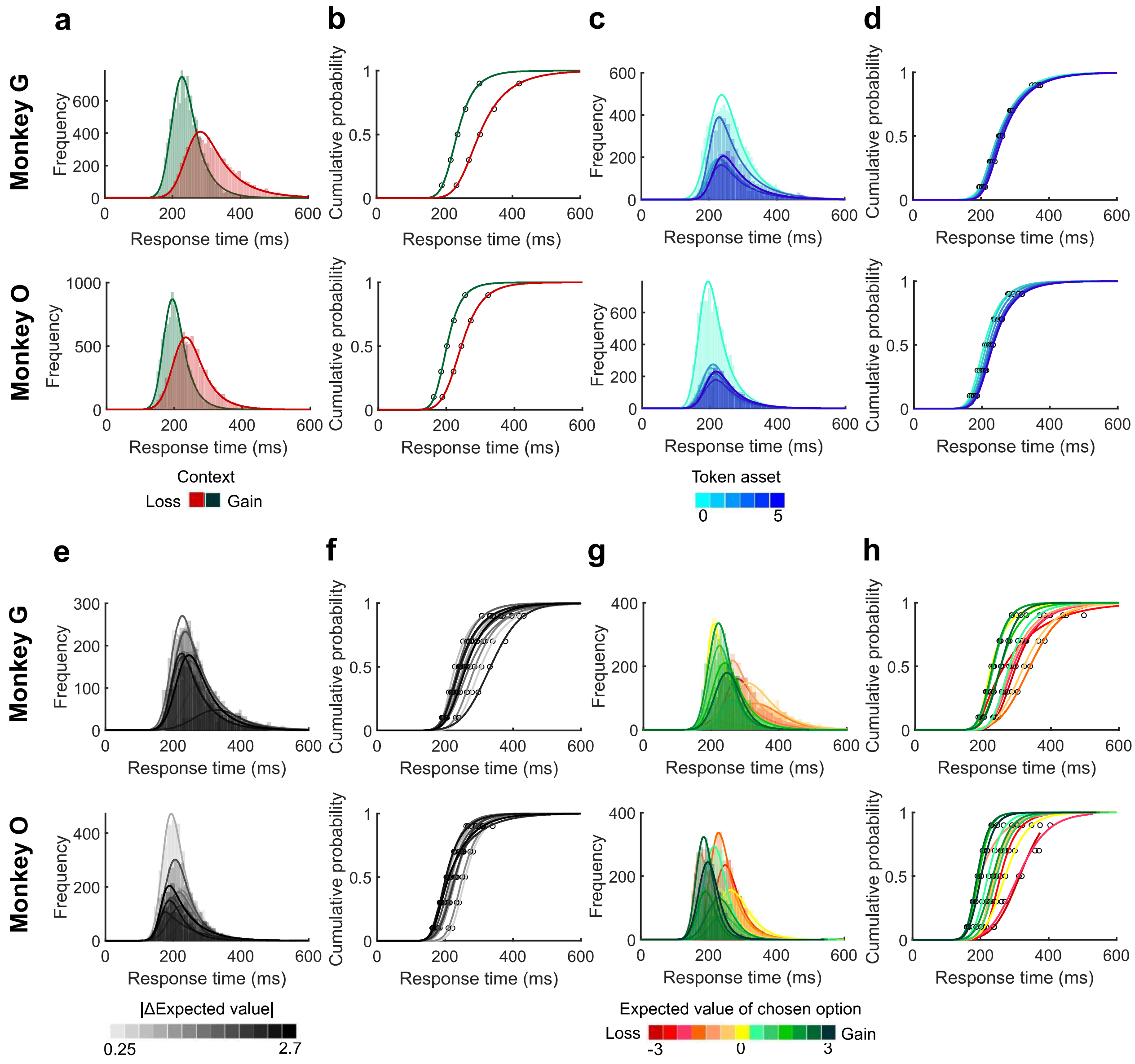

Supplementary Fig. 3. Response time of monkey's choice.

a. Distribution of response times when monkeys made decisions in the gain (green) and loss (red) context for each monkey. Histograms with light color indicate the raw data distributions and curves with dark color indicate the best-fitting (ex-Gaussian) distributions.

b. Cumulative distribution function (CDF) of response times in the gain (green) and loss (red) context.

c. Distribution of response times when monkeys made decisions with different start token numbers. The color gradients from light to dark blue indicate token number from 0 to 5.

d. CDF of response times with different start token numbers.

e. Distribution of response times when monkeys made decisions with different absolute values of expected value difference between gamble and sure option (|ΔEVgs|s). The color gradients indicate |ΔEVgs| from small to large. One monkey took more time to make a choice when the difference expected value between options was small (regression analysis; monkey O: $\beta_{RT\_|\Delta EVgs|}$= -5.11, $p < 10^{-3}$), indicating a task-difficulty dependent response time. However, the other monkey showed no significant effect of this variable (regression analysis; monkey G: $\beta_{RT\_|\Delta EVgs|}$= -0.50, p = 0.74).

f. CDF of response times with different |ΔEVgs|s.

g. Distribution of response times with different expected values of the chosen option (ChV). The color gradients indicate chosen values from small to large. Both monkeys made faster choices as the expected value of the chosen option increased (regression analysis; monkey G: β_{RT_ChV} = 2.83, p = 0.19; monkey O: β_{RT_ChV}= 3.50, $p < 10^{-2}$). This likely reflects an elevated motivation for high-value options.

h. CDF of response times with different EVs of chosen option.

n = 37 sessions for both monkey G and monkey O.

Supplementary Fig. 4

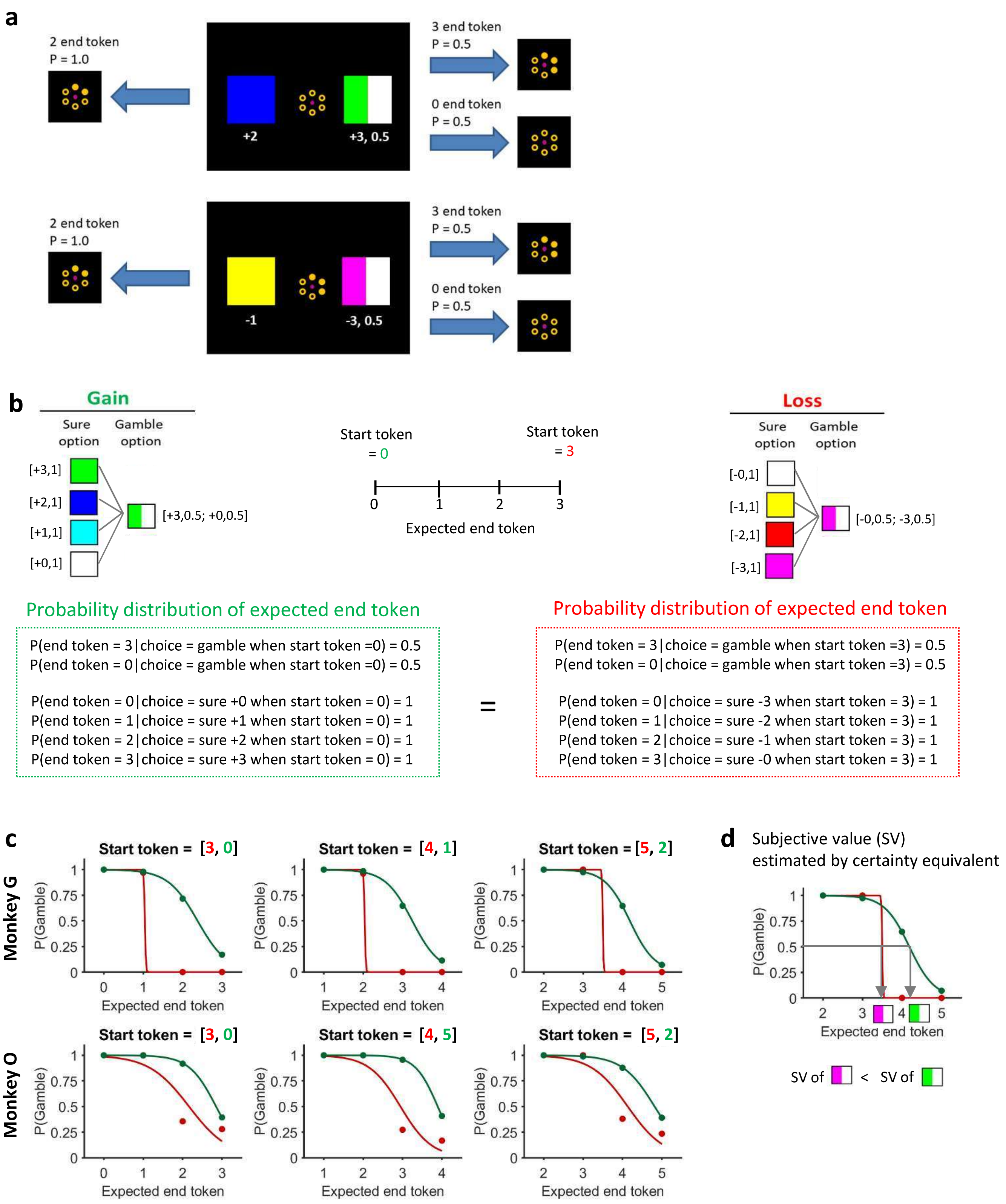

Supplementary Fig. 4. Monkeys' risk preferences changed across gain/loss contexts with different start token numbers cannot be attributed to their evaluation of each available option based on the expected final token number

Prospect theory implies the use of a 'relative value' framework, where the value of an outcome depends on the change in assets relative to a reference point. Alternatively, the monkeys could use an 'absolute value' framework, where the value of an outcome depends on the final asset number. To test, which of these value frameworks is used by the monkeys, we compared trials with the same outcome in terms of final token number, but which resulted from either gaining or losing tokens, because the start token number was different.

a. For example, consider a trial with a start token number of 0, in which a gamble option with an equal probability of gaining 3 or 0 tokens is offered versus a sure option of gaining 2 tokens. The expected end token outcomes of this trial (owning 3 or 0 tokens, each with p=0.5 versus owning 2 tokens with p=1) are identical to a trial with a start token number of 3, in which a gamble option with an equal probabilityof losing 3 or 0 tokens is offered versus a sure option of losing 1 token.

b. The same two gambles can be systematically matched with other pairs of sure options that reached the same final token number by either gaining or losing tokens. The dotted boxes show the probability distribution of expected end token numbers (i.e., the possible final token numbers and their probability) for each of the gambles and a set of corresponding sure options. The probability distribution of expected end token number for gain trials at a start token number of 0 (green dotted box) is identical to the one for loss trials at a start token number of 3 (red dotted box). Thus, the subjective value of the two gamble options should be identical in an absolute value framework, but different in a relative value framework.

c. Choice functions indicate the probability of choosing the gamble option as a function of expected end token number for the paired sure options. The expected end token number is a function of the indicated change in number of token and the start token number (e.g., expected end token number is 2 when choosing sure option +2 at start token number 0 or when choosing sure option -1 at start token number 3). The choice function computed from the gain trials is indicated in green and the one computed from the loss trials is indicated in red. For each monkey, three corresponding choice functions are shown for two identical sets of gamble and sure pairs that are presented for three corresponding start token settings (indicated on top of the choice functions).

d. The choice functions allow us to estimate the subjective value (SV) of the corresponding gamble option in units of value associated with the paired sure options (here: 'expected end token number') using the model-free certainty equivalent method. The probability of the monkey to choose a gamble depends on the difference between its own value and the value of the alternative sure option. Whenthe value of the sure option is small, monkeys are more likely to choose the gamble. As the sure option'svalue increases, monkeys increasingly choose the sure option. The choice function allows us to estimate the point when the probability of choosing either the gamble or the sure option are equal [P(G) = 0.5].At this point, the subjective value of the two options must be equal, independent of the underlying utility functions that relate value to physical outcome. Therefore, the subjective value of the gamble is equivalent to the corresponding sure option value at this indifference point. This value is referred to as the certainty equivalent (CE) [Luce, R. D. *Utility of gains and losses: Measurement-theoretical and experimental approaches*. (Psychology Press, 2014)]. The grey arrows indicate the two CE values. Their large difference shows that the SVs of the two gamble options in gain and loss trials are different, even so their outcomes are the same in an absolute value framework.

Supplementary Fig. 5

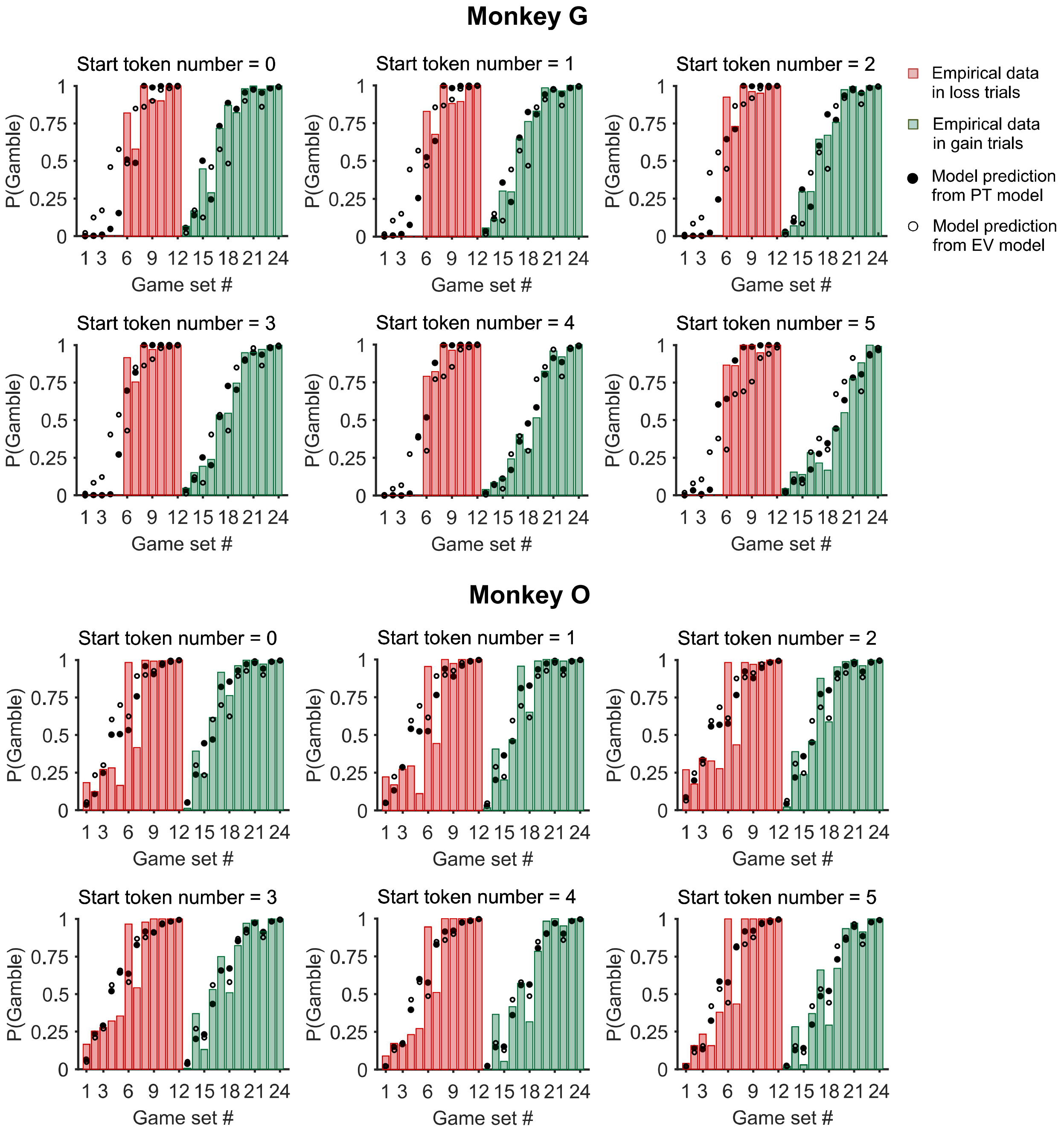

Gamble set (Gamble vs. Sure)

1: [-0, 0.10; -3, 0.90] vs. [-0, 1]	13: [+3, 0.10; +0, 0.90] vs. [+3, 1]
2: [-0, 0.50; -3, 0.50] vs. [-1, 1]	14: [+3, 0.50; +0, 0.50] vs. [+3, 1]
3: [-0, 0.50; -3, 0.50] vs. [-0, 1]	15: [+3, 0.10; +0, 0.90] vs. [+2, 1]
4: [-0, 0.75; -3, 0.25] vs. [-0, 1]	16: [+3, 0.75; +0, 0.25] vs. [+3, 1]
5: [-0, 0.50; -3, 0.50] vs. [-1, 1]	17: [+3, 0.50; +0, 0.50] vs. [+2, 1]
6: [-0, 0.10; -3, 0.90] vs. [-2, 1]	18: [+3, 0.10; +0, 0.90] vs. [+1, 1]
7: [-0, 0.75; -3, 0.25] vs. [-1, 1]	19: [+3, 0.75; +0, 0.25] vs. [+2, 1]
8: [-0, 0.10; -3, 0.90] vs. [-3, 1]	20: [+3, 0.50; +0, 0.10] vs. [+1, 1]
9: [-0, 0.50; -3, 0.50] vs. [-2, 1]	21: [+3, 0.75; +0, 0.25] vs. [+1, 1]
10: [-0, 0.75; -3, 0.25] vs. [-2, 1]	22: [+3, 0.10; +0, 0.90] vs. [+0, 1]
11: [-0, 0.50; -3, 0.50] vs. [-3, 1]	23: [+3, 0.50; +0, 0.50] vs. [+0, 1]
12: [-0, 0.75; -3, 0.25] vs. [-3, 1]	24: [+3, 0.75; +0, 0.25] vs. [+0, 1]

Supplementary Fig. 5. Behavioral results and model simulations.

The probability of choosing the gamble option, P(Gamble), for all possible choice target pairs (the gamble set), separately for all possible token assets ranging from 0-5. The gamble set consists of 12 different pairs of gamble and sure options in the gain context, and another 12 pairs of gamble and sure options in the loss context (see list below). Colored bars represent the actual choice data (gain in green and loss in red). Black (prospect theory model) and white (expected value model) dots represent the model predictions. Bars are sorted according to ΔEV (EV gamble - EV sure) from small to large in loss and gain context, respectively. The top plots show behavior and model predictions for monkey G and the bottom plots for monkey O.

Supplementary Fig. 6

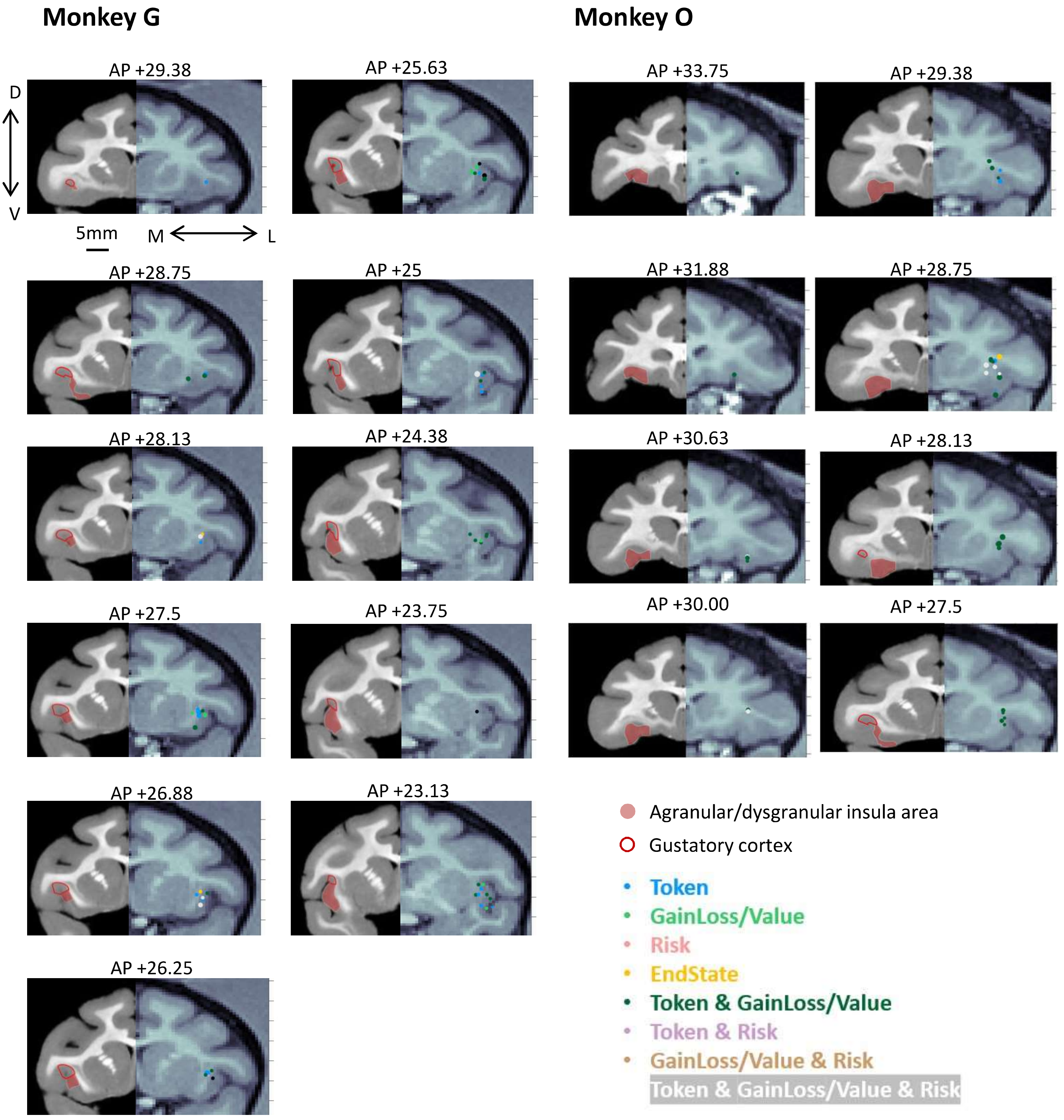

Supplementary Fig. 6. Recording sites with location of neurons of different functional types. Coronal MRI sections for each monkey show the locations of recorded neurons. The right side of each section shows the MRI from the anatomical scan of each monkey performed before surgery. Superimposed on each section is the estimated location of each recorded neuron based on penetration coordinates and recording depth. Neuronal classification according to the regression model is marked in different colors. The dot size indicates the number of units recorded in the location. Different colors indicate different functional signals encoded by the neurons. The position of each section in stereotactic coordinates is indicated on top. The left side of each section shows the most similar section in the macaque brain atlas of Saleem and Logothetis (2012). The location of the agranular and dysgranular insula (filled pink area), and gustatory cortex (red outlined area) are indicated in each section.

Supplementary Fig. 7

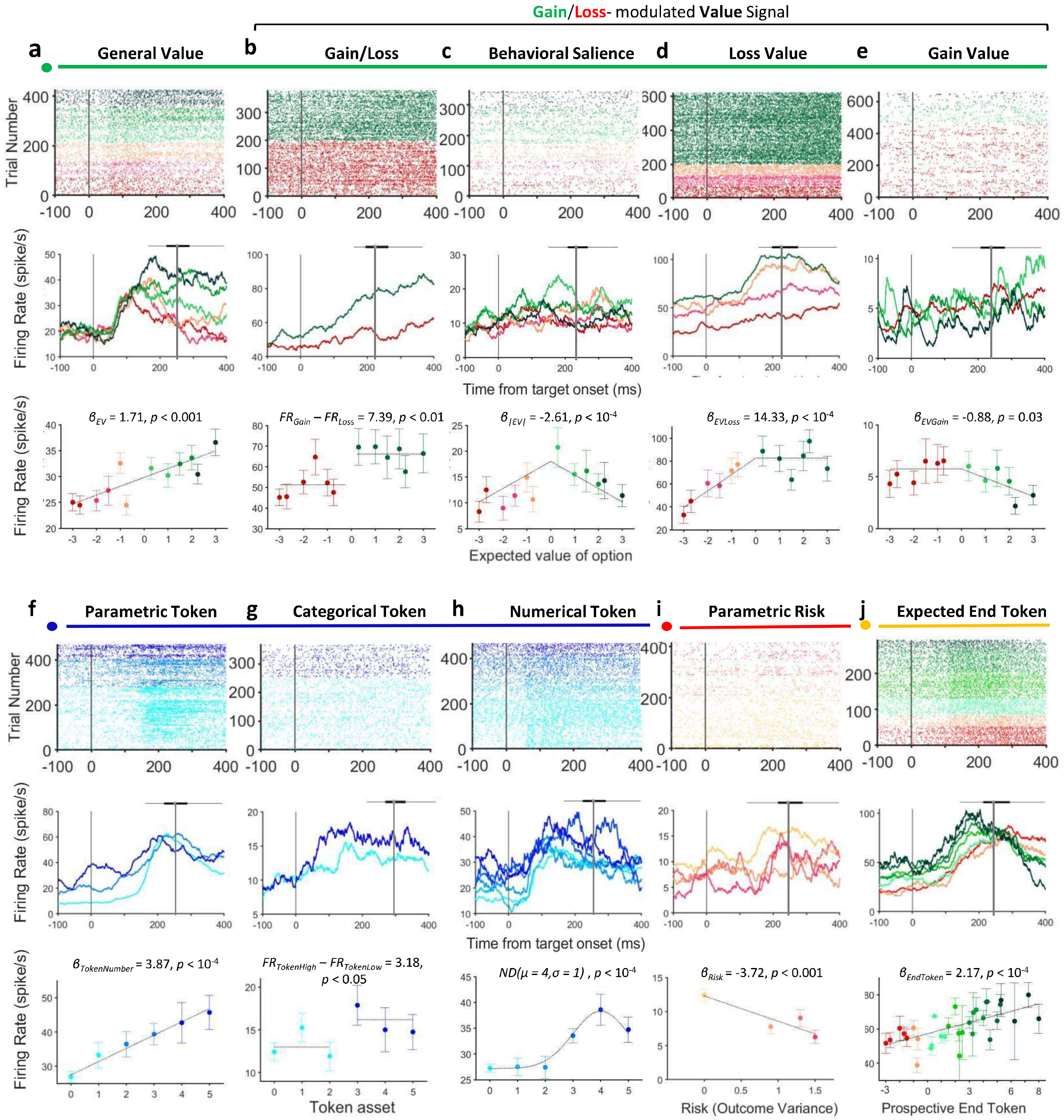

Supplementary Fig. 7. Raster plot, spike density function, and mean firing rate in the choice period of example neurons shown in figure 3c-l.

Example neuron showing: **a.** General value signal: monotonic encoding of value across gain/loss contexts; **b.** Gain/Loss value signal: categorical encoding of context of gain and loss; **c.** Behavioral salience signal: monotonic encoding of value in gain and loss context but with inverse directions; **d.** Loss value signal: encoding of value only in the loss context; **e.** Gain value signal: encoding of the value only in the gain context; **f.** Parametric token signal: monotonic encoding of the start token number; **g.** Categorical token signal: categorical encoding of the start token number in low and high level; **h.** Numerical token signal: neuronal response tunes to a specific number of start token (=4); **i.** Parametric risk signal: monotonic encoding of risk; **j.** Expected end token signal: monotonic encoding of expected end token number.

a-j. Upper, middle, and lower panels indicate rater plot, spike density function (SDF), and mean firing rate of each example neuron at different levels of specific task-related variable aligned by the target onset (t=0), respectively. Mean firing rate (presented as mean values ± SEM) was calculated using the window from target onset to saccade initiation, which varied across trials. The distribution of saccade onset was presented as a boxplot on top of each SDF. The box plot indicates median (vertical middle line), 25th, 75th percentile (box) and 5th and 95th percentile (whiskers). For clarity, when plotting the raster plot and SDF, data of some EV levels were grouped together, as indicated by the color codes.

Supplementary Fig. 8

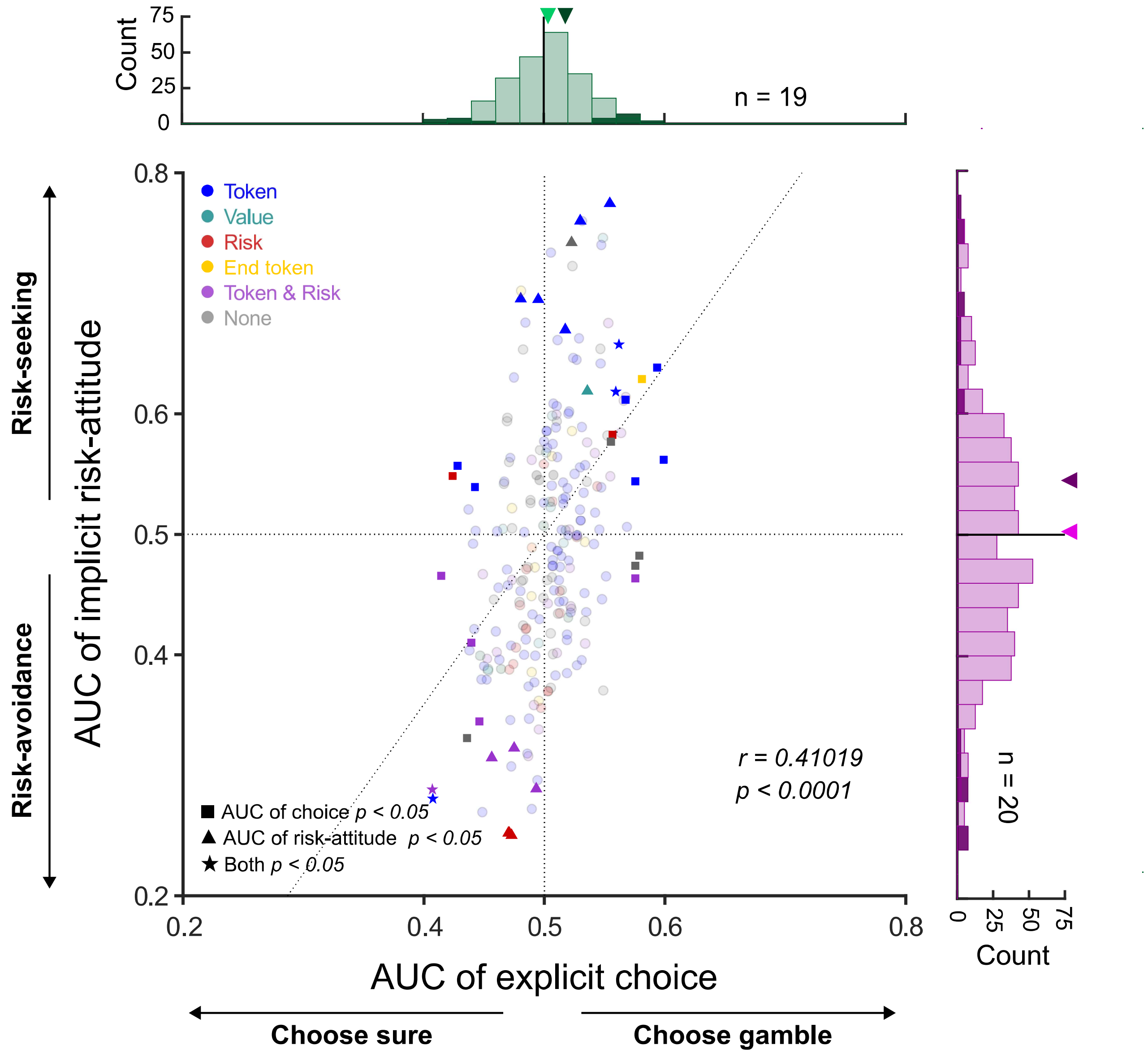

Supplementary Fig. 8. Distribution of area under the curve (AUC) of receiver operating characteristic (ROC) for explicit choice and implicit risk-attitude in neurons encode different kind of decision-related signals.

AUC values capturing the covariation of each neuron with differences in explicit choice (choosing gamble or sure) and implicit risk-attitude (risk-seeking or risk-avoidance). Each point represents one neuron (n = 240). The color of each point indicates the functional type of signal encoded by the neuron, while the shape indicates the significance of the two AUC values. The vertical and horizontal dashed lines show the area of no significant discrimination ability (AUC of choice = 0.5 and AUC of risk-attitude = 0.5). The hatched line represents the linear regression relating the AUC of choice and AUC of risk-attitude (*r* and *p* values refer to the regression slope). In the marginal distributions, neurons with significant AUC values are indicted in darker shades. The number of significant neurons is listed for each distribution. The arrowheads indicate the average AUC values across the entire distribution (light green or light purple) and the average AUC of the subset of neurons with significant values (dark green or dark purple), respectively.

Supplementary Table 1. Summary of the number and percentage of significant responding neurons in different subsets of neuron types for AIC neurons recorded from each monkey.

Gain/Loss-Value	Gain/Loss	Loss Value	Gain Value	Behavioral Salience	General Value				All
Monkey G (n=142)<br>Monkey O (n=98)	4<br>(3%)<br>9<br>(9%)	9<br>(6%)<br>20<br>(20%)	1<br>(1%)<br>3<br>(3%)	4<br>(3%)<br>9<br>(9%)	3<br>(2%)<br>11<br>(11%)				21<br>(15%)<br>52<br>(53%)
Token	Parametric Token	Categorical Token	Token 0	Token 1	Token 2	Token 3	Token 4	Token 5	All
Monkey G (n=142)<br>Monkey O (n=98)	7<br>(5%)<br>5<br>(5%)	9<br>(6%)<br>2<br>(2%)	2<br>(1%)<br>9<br>(9%)	9<br>(6%)<br>7<br>(7%)	7<br>(5%)<br>14<br>(14%)	6<br>(4%)<br>6<br>(6%)	4<br>(3%)<br>6<br>(6%)	4<br>(3%)<br>4<br>(4%)	48<br>(34%)<br>54<br>(55%)
Risk	Parametric Risk	Categorical Risk							All
Monkey G (n=142)<br>Monkey O (n=98)	4<br>(3%)<br>5<br>(5%)	6<br>(4%)<br>4<br>(4%)							10<br>(7%)<br>9<br>(9%)
Expected End Token	End Token								All
Monkey G (n=142)<br>Monkey O (n=98)	6<br>(4%)<br>3<br>(3%)								6<br>(4%)<br>3<br>(3%)

Supplementary Table 2. Summary of the number and percentage of neurons positively or negatively correlated to different decision-related variables.

Gain/Loss-Value	Gain/Loss	Loss Value	Gain Value	Behavioral Salience	General Value				All
Positive correlation<br>Negative correlation	8<br>(62%)<br>5<br>(38%)	27<br>(93%)<br>2<br>(7%)	0<br>(0%)<br>4<br>(100%)	0<br>(0%)<br>13<br>(100%)	13<br>(93%)<br>1<br>(7%)				48<br>(66%)<br>25<br>(34%)
Token	Parametric Token	Categorical Token	Token 0	Token 1	Token 2	Token 3	Token 4	Token 5	All
Positive correlation<br>Negative correlation	11<br>(85%)<br>2<br>(15%)	4<br>(36%)<br>7<br>(64%)	0<br>(0%)<br>11<br>(100%)	8<br>(50%)<br>8<br>(50%)	21<br>(88%)<br>3<br>(12%)	12<br>(100%)<br>0<br>(0%)	8<br>(80%)<br>2<br>(20%)	5<br>(63%)<br>3<br>(37%)	69<br>(66%)<br>36<br>(34%)
Risk	Parametric Risk	Categorical Risk							All
Positive correlation<br>Negative correlation	3<br>(33%)<br>6<br>(64%)	3<br>(30%)<br>7<br>(70%)							6<br>(32%)<br>13<br>(68%)
Expected End Token	End Token								All
Positive correlation<br>Negative correlation	5<br>(56%)<br>4<br>(44%)								5<br>(56%)<br>4<br>(44%)
