# Supplementary material for: Primate anterior insular cortex represents economic decision variables proposed by prospect theory
Source: Nat Commun. 2022 Feb 7;13:717. doi: 10.1038/s41467-022-28278-9 (PMC8821715; doi:10.1038/s41467-022-28278-9)
Supplement: Supplementary file 3 — Reporting Summary [file 41467_2022_28278_MOESM3_ESM.pdf]

nature research

Corresponding author(s): Veit Stuphorn

Last updated by author(s): Nov 29, 2021

Reporting Summary

Nature Research wishes to improve the reproducibility of the work that we publish. This form provides structure for consistency and transparency in reporting. For further information on Nature Research policies, see our Editorial Policies and the Editorial Policy Checklist.

Statistics

For all statistical analyses, confirm that the following items are present in the figure legend, table legend, main text, or Methods section.

n/a	Confirmed	
☐	☒	The exact sample size (n) for each experimental group/condition, given as a discrete number and unit of measurement
☐	☒	A statement on whether measurements were taken from distinct samples or whether the same sample was measured repeatedly
☐	☒	The statistical test(s) used AND whether they are one- or two-sided<br>*Only common tests should be described solely by name; describe more complex techniques in the Methods section.*
☐	☒	A description of all covariates tested
☐	☒	A description of any assumptions or corrections, such as tests of normality and adjustment for multiple comparisons
☐	☒	A full description of the statistical parameters including central tendency (e.g. means) or other basic estimates (e.g. regression coefficient) AND variation (e.g. standard deviation) or associated estimates of uncertainty (e.g. confidence intervals)
☒	☐	For null hypothesis testing, the test statistic (e.g. F, t, r) with confidence intervals, effect sizes, degrees of freedom and P value noted<br>*Give P values as exact values whenever suitable.*
☒	☐	For Bayesian analysis, information on the choice of priors and Markov chain Monte Carlo settings
☒	☐	For hierarchical and complex designs, identification of the appropriate level for tests and full reporting of outcomes
☒	☐	Estimates of effect sizes (e.g. Cohen's d, Pearson's r), indicating how they were calculated

Our web collection on statistics for biologists contains articles on many of the points above.

Software and code

Policy information about availability of computer code

Data collection	Monkeylogic software (https://www.brown.edu/Research/monkeylogic/) was used to control task events, stimuli, and reward, as well as monitor and store behavioral events. Eye positions were monitored with an infrared corneal reflection system, EyeLink 1000 (SR Research) at a sampling rate of 1000 Hz. Single neuron activities were recorded extracellularly with single tungsten microelectrodes (impedance of 2-4 MΩs, Frederick Haer, Bowdoinham, ME). Electrophysiological data were collected using the TDT system (Tucker & Davis).
Data analysis	All analyses were performed using self-written Matlab (MathWorks, R2018b) code.

For manuscripts utilizing custom algorithms or software that are central to the research but not yet described in published literature, software must be made available to editors and reviewers. We strongly encourage code deposition in a community repository (e.g. GitHub). See the Nature Research guidelines for submitting code & software for further information.

Data

Policy information about availability of data

All manuscripts must include a data availability statement. This statement should provide the following information, where applicable:

- Accession codes, unique identifiers, or web links for publicly available datasets
- A list of figures that have associated raw data
- A description of any restrictions on data availability

Data and software are available upon request.

April 2020

Field-specific reporting

Please select the one below that is the best fit for your research. If you are not sure, read the appropriate sections before making your selection.

☒ Life sciences ☐ Behavioural & social sciences ☐ Ecological, evolutionary & environmental sciences

For a reference copy of the document with all sections, see nature.com/documents/nr-reporting-summary-flat.pdf

Life sciences study design

All studies must disclose on these points even when the disclosure is negative.

Sample size	Two male rhesus monkeys were trained to perform a token-based gambling task in this study.<br>37 behavioral sessions were included for each monkey.<br>240 neurons (142 and 98 for the two monkeys, respectively) were reported in this study.
Data exclusions	All behavioral sessions with corresponding neural data were included.<br>Most behavioral analyses (except the fixation break analysis) and behavioral modeling included only the completed trials.<br>Neuronal correlates of decision-related variables (figure 2 and 3) were analyzed with no-choice trials.<br>AUROC analysis (figure 4) were analyzed with choice trials.
Replication	Not applicable.
Randomization	The choice and force choice trials were pseudo-randomly interleaved in blocks so that each block consisted of all 24 choice trials and 13 n0-choice trials.
Blinding	Not applicable.

Reporting for specific materials, systems and methods

We require information from authors about some types of materials, experimental systems and methods used in many studies. Here, indicate whether each material, system or method listed is relevant to your study. If you are not sure if a list item applies to your research, read the appropriate section before selecting a response.

Materials & experimental systems

n/a	Involved in the study	
☒	☐	Antibodies
☒	☐	Eukaryotic cell lines
☒	☐	Palaeontology and archaeology
☐	☒	Animals and other organisms
☒	☐	Human research participants
☒	☐	Clinical data
☒	☐	Dual use research of concern

Methods

n/a	Involved in the study	
☒	☐	ChIP-seq
☒	☐	Flow cytometry
☒	☐	MRI-based neuroimaging

Animals and other organisms

Policy information about studies involving animals; ARRIVE guidelines recommended for reporting animal research

Laboratory animals	Two male rhesus monkeys (Monkey G: 7.2 kg, Monkey O: 9.5 kg)
Wild animals	N/A
Field-collected samples	N/A
Ethics oversight	All animal care and experimental procedures were conducted in accordance with the US public Health Service policy on the humane care and use of laboratory animals and were approved by the Johns Hopkins University Institutional Animal Care and Use Committee (IACUC).

Note that full information on the approval of the study protocol must also be provided in the manuscript.
